# Chikunguña: primera arbovirosis emergente en el siglo XXI en las Américas

**DOI:** 10.26633/RPSP.2017.108

**Published:** 2017-06-06

**Affiliations:** 1 Director, Enfermedades Transmisibles y Análisis de Salud Organización Panamericana de la Salud Washington, D.C. Unidos de América Director, Enfermedades Transmisibles y Análisis de Salud, Organización Panamericana de la Salud, Washington, D.C., Estados Unidos de América

En diciembre del 2013, el panorama de las arbovirosis cambió para siempre en el Hemisferio Occidental. Esa es la fecha de la primera notificación de circulación autóctona del virus chikunguña en la Región de las Américas, en la isla de Saint Martin (1). A partir de ese momento, la epidemia se extendió a una velocidad sin precedentes en la Región. La expansión fue inesperada en el número de casos, la diseminación geográfica, el desafío a los servicios de salud, la letalidad y el impacto económico. Los programas de control de vectores, acostumbrados a trabajar con el dengue, tuvieron que reactivarse para hacer frente a una nueva amenaza.

El conocimiento sobre el virus chikunguña (virología, fisiopatología, manifestaciones clínicas graves, transmisión vertical, manifestaciones crónicas y secuelas e impacto económico) se comenzó a desarrollar a partir de los brotes en los territorios franceses en el Pacífico, y en la India en el 2007. Sin embargo, la falta de evidencia sobre el comportamiento clínico en las Américas motivó un gran esfuerzo por parte de la comunidad científica y de salud pública para desarrollar y publicar sus experiencias y conocimientos.

El análisis molecular del virus que circula en las Américas revela que está relacionado filogenéticamente al genotipo asiático (2), uno de los tres genotipos descritos, junto con el de África del Oeste (WA) y el Este-Central-Sur Africano (ECSA). La diseminación del virus fue explosiva; en el primer año de circulación se contabilizaron más de un millón de casos sospechosos y confirmados de chikunguña (3). Los sistemas de vigilancia epidemiológica hubieron de adaptar sus sistemas para informar sobre la magnitud y dinámica de la expansión del virus chikunguña pero algunos aspectos del monitoreo quedaron pendientes, tales como la notificación de casos atípicos, graves y fallecidos relacionados con dicha infección. La letalidad reportada por los sistemas de vigilancia epidemiológica posiblemente subestimó el efecto del virus en la población; quizás esto se haga evidente al analizar de manera retrospectiva los datos de mortalidad y comprobar si durante el paso del virus en la Región hubo un incremento de la mortalidad de los grupos de edad avanzada (mayores de 65 años).

## Una arbovirosis desconocida en las Américas

Conocer los factores que condujeron a la rápida expansión geográfica del virus chikunguña en las Américas resulta una prioridad. Entre estos factores se consideran la falta de inmunidad de la población —que nunca antes había estado expuesta a este virus—, la viremia elevada y prolongada —que aumenta las posibilidades de transmisión—, así como determinantes ambientales y sociales, como altas temperatura, humedad, vegetación, lluvias y densidad poblacional que favorecen la proliferación de mosquitos.

La complejidad de la vigilancia y el control de las arbovirosis, inclusive el dengue, se incrementó significativamente desde la introducción del virus chikunguña en el 2013 y del virus del Zika en el 2014–2015. Para estas enfermedades se requiere un abordaje integrado, en el que los aspectos relacionados con la atención clínica, la plataforma de laboratorio y el control de vectores resultan esenciales.

El reconocimiento de la presencia de cuadros clínicos compatibles con infección por chikunguña es esencial para alertar de manera precoz sobre la circulación del virus, sin olvidar la realización de un diagnóstico diferencial que garantice el adecuado manejo de enfermedades que requieran un tratamiento específico (por ej., dengue, leptospirosis o sepsis de origen bacteriano). Los retos que plantea una epidemia de chikunguña no solo se centran en la respuesta en la fase aguda, sino en la atención de las formas subagudas y crónicas, que afectan la calidad de vida y el bienestar de las personas.

El laboratorio de virología resulta esencial desde el punto de vista de salud pública para confirmar la circulación del virus y las muertes relacionadas con el chikunguña, así como desde el punto de vista clínico para el diagnóstico de las formas atípicas y graves y la transmisión maternoinfantil. Los países desarrollaron rápidamente su capacidad de laboratorio para detectar a este nuevo virus patógeno.

La rápida diseminación del chikunguña fue un claro indicador de que las estrategias de control de vectores, tal como hoy se aplican, resultaron insuficientes para la contención de las arbovirosis. Se conocía que la extensa diseminación del vector en las Américas (*Aedes aegypti* y *Ae. albopictus*), relacionada con diferentes factores sociales y económicos, así como con el cambio climático y los ecosistemas en el nivel local y global, constituye el mayor reto para lograr el control de las arbovirosis en la Región, a pesar del compromiso de los países para la implementación progresiva del Manejo Integrado de Vectores (4). Es preciso incorporar elementos procedentes de la caracterización ecológica así como, de manera progresiva, nuevas tecnologías con resultados probados.

La complejidad creciente del abordaje de las arbovirosis desde una perspectiva de salud pública motivó la presentación y aprobación de una estrategia regional sobre arbovirosis en el 55° Consejo Directivo de la Organización Panamericana de la Salud (OPS) (5). En ella, los países se comprometieron a dar una respuesta integrada a los aspectos comunes sobre la atención clínica de las arbovirosis, la necesidad de una red de laboratorios, la vigilancia epidemiológica y el control de vectores.

## Hacia un manejo integrado de las arbovirosis

El continente americano tiene una ecología que permite la endemia de enfermedades metaxénicas, en particular en su amplia área intertropical. Las enfermedades transmitidas por vectores están ligadas a los orígenes de la OPS. En 1902, representantes de 11 países de las Américas se reunieron para hacer frente a la malaria y la fiebre amarilla en la primera Convención General Internacional Sanitaria de las Repúblicas Americanas que, con el devenir del tiempo, se convertirá en la OPS. Ciento quince años después, la OPS sigue brindando apoyo técnico a los países para hacer frente a estas y nuevas enfermedades trasmitidas por vectores. Así, las lecciones aprendidas en la respuesta frente al chikunguña fueron de gran valor en mayo del 2015 cuando se confirmó la circulación del virus del Zika en Brasil. Los servicios de salud, el control de vectores y la capacidad de laboratorio de los países disponían de la capacidad de adaptación y respuesta a un nuevo desafío.

Sin embargo, persisten brechas de evidencia en relación con el chikunguña, y quedan pendientes varios aspectos de conocimiento científico con impacto en la salud pública. Indudablemente, se ha de monitorear el comportamiento del virus en las Américas, no solo en las poblaciones humanas, sino también en los medios silvestres. La abundancia de especies de primates en muchos de los países, así como de mosquitos que nunca han sido expuestos al chikunguña, pueden brindar oportunidades para que este virus establezca ciclos selváticos que hasta la fecha no se han documentado fuera de África (6). Otros aspectos pendientes son la evaluación del impacto en la mortalidad poblacional en adultos mayores, un conocimiento exacto en la carga en los sistemas de salud, la estimación de la carga de enfermedad y el cálculo del costo económico para los países.

Este número especial de la *Revista Panamericana de Salud Pública* tiene como objetivo ayudar a cerrar algunas de estas brechas de evidencia, y brinda una plataforma para la diseminación de los resultados de estudios de investigación relevantes desde el punto de vista de la salud pública en las Américas.

## Agradecimiento

La *Revista Panamericana de Salud Pública* agradece las contribuciones de los miembros del Comité Editorial. Sus contribuciones y su dedicación a este Número especial sobre chikunguña en la Región de las Américas ayudaron a hacer los manuscritos más interesantes, más precisos y más útiles para nuestros lectores y los profesionales que trabajan para mejorar la salud de los pueblos de las Américas.

La Revista agradece especialmente a los Centros para el Control y la Prevención de Enfermedades de los Estados Unidos, cuyo apoyo financiero y programático fue esencial para la publicación este número especial.

Sylvain Aldighieri, Pan American Health Organization/World Health Organization, United States of America

Virgen Gómez, Hospital Infantil Dr. Robert Reid Cabral, Santo Domingo, República Dominicana

José Moya, Organización Panamericana de la Salud/Organización Mundial de la Salud, Argentina

Roger Nasci, North Shore Mosquito Abatement District, Northfield, Illinois, United States of America

Karen Polson-Edwards, Caribbean Public Health Agency (CARPHA), Trinidad and Tobago

Ernesto Pleités, Instituto Nacional de Salud/Ministerio de Salud, El Salvador

Ann Powers, Centers for Disease Control and Prevention, United States of America

Pilar Ramón-Pardo, Pan American Health Organization/World Health Organization, United States of America

Fabrice Simon, Hôpital Alphonse Laveran, France

Jaime Torres, Instituto de Medicina Tropical, Venezuela

Pedro Fernando da Costa Vasconcelos, Instituto Evandro Chagas/Ministério da Saúde, Brasil

Sergio Yactayo, World Health Organization, Switzerland
